# Large feet are beneficial for eiders *Somateria mollissima*

**DOI:** 10.1002/ece3.5384

**Published:** 2019-07-21

**Authors:** Anders Pape Møller, Karsten Laursen

**Affiliations:** ^1^ Ecologie Systématique Evolution Université Paris‐Sud Orsay Cedex France; ^2^ CNRS, AgroParisTech Université Paris‐Saclay Orsay Cedex France; ^3^ Ministry of Education Key Laboratory for Biodiversity Science and Ecological Engineering, College of Life Sciences Beijing Normal University Beijing China; ^4^ Department of Bioscience Aarhus University Rønde Denmark

**Keywords:** condition, duck's feet, eider, locomotion, uropygial gland, webbed feet

## Abstract

1. Many waterbirds have fully (totipalmate) or partially webbed (palmate) feet that are used for locomotion in aquatic environments.
2. If webbed feet and wings both contribute to efficient diving, we predicted a positive association between the area of webbed feet and the size of the frontal locomotor apparatus (wing area, heart mass, and breast muscle, after adjusting for any partial effects of body size). We predicted that individuals able to acquire more and better quality food due to larger webbed feet should have larger livers with higher concentrations of fat‐soluble antioxidants such as vitamin E, and invest more in immune function as reflected by the relative size of the uropygial gland than individuals with small webbed feet.
3. Here, we examine if the area of webbed feet is correlated with locomotion, diet, and body condition in a sea‐duck, the eider (*Somateria mollissima*). We analyzed an extensive database of 233 eiders shot in Danish waters and at Åland, Finland during winter and early spring.
4. Eiders with larger webbed feet had a larger locomotor apparatus, but did not have larger body size, they had larger uropygial glands that waterproof the plumage, they had larger beak volume and larger gizzards, and they had higher body condition.
5. These findings imply that eiders with large webbed feet benefitted in terms of locomotion, feeding, and reproduction.

## 1 INTRODUCTION

Birds vary greatly in foot size and shape relating to locomotion, feeding, and reproduction (Elphick, Dunning, Jack, & Sibley, 2001; Kochan, 1994; Proctor & Lynch, 1993). Legs and feet of birds play important roles in walking (most species), running (ostriches, grouse, turkeys), climbing (woodpeckers, nuthatches, tree‐creepers), swimming and steering underwater (ducks, grebes, loons), perching (as on a branch) or clinging (Elphick et al., 2001; Kochan, 1994; Proctor & Lynch, 1993). Other functions include carrying (like ospreys *Pandion haliaetus* holding fish) and flight‐related locomotion (Elphick et al., 2001; Kochan, 1994; Proctor & Lynch, 1993). Feeding and related functions of feet include capture and killing of prey and pulling apart food (with help from the bill). Some grebes may even run on water for mate acquisition (Clifton, Hedrick, & Biewener, 2015).

Webbed feet are characteristic features of bird species living in aquatic habitats (Elphick et al., 2001; Kochan, 1994; Proctor & Lynch, 1993). Sea ducks rely on both webbed feet and wings for diving, sweeping their webbed feet backwards (Richman & Lovvorn, 2008). Loons *Gavia immer* swim by paddling their feet laterally as in grebes and cormorants (Clifton & Biewener, 2018). Eiders use both wing and foot propulsion during descent. The power face of foot strokes coincides with the transition between upstroke and downstroke of the wings (Heath, Gilchrist, & Ydenberg, 2006). The delta shape of their webbed feet is able to generate propulsive forces from drag at the start of the stroke of the feet to the lift later in the stroke (Johansson & Norberg, 2003). Some sea ducks use both feet and wings to swim (Richman & Lovvorn, 2008). The use of both feet and wings increased bottom duration by 59% compared to dives with feet only with potential consequences for ingestion rate (Richman & Lovvorn, 2008).

Webbed feet are often large, and they show considerable variation in size among individuals (A. P. Møller pers. obs.), raising the question what is the basis for such variation. Surprisingly, there is no publication dealing with the consequences of intraspecific variation in size of webbed feet, and this deficit prompted our study. If large feet are beneficial, we assume that a larger foot size is associated with a larger locomotor apparatus such as a larger wing area and a larger heart. Although there is a genetic basis for the size of webbed feet (Ganan, Macias, Basco, Merino, & Hurle, 1998), and the varied and important roles that feet play across bird species, there is currently a significant lack of understanding of the functional significance of foot size.

Eiders are well‐known capital breeders (relying on stored food throughout the incubation period) that build up body reserves during winter for subsequent breeding in spring and early summer (Meijer & Drent, 1999). Female eiders arrive during spring entering colonies and producing a clutch of 4–5 eggs that are incubated for about 26 days. The female sits on the nest during this period without feeding. Therefore, females must build up sufficient body reserves for breeding at the wintering grounds and off the breeding grounds (Hobson, Jaatinen, & Öst, 2015). During winter, eiders are located off shore in large flocks feeding on benthos at 4–8 m depth, with a range of 0–20 m (Petersen et al., 2010). In the prebreeding period, they stay near the shore feeding in shallow water. Studies in the Wadden Sea have shown that the number of eiders increases with mussel stocks, and individuals feeding on mussels have superior body condition than those taking other types of prey, which makes it attractive for eiders to feed on mussel beds (Laursen, Asferg, Frikke, & Sunde, 2009; Laursen & Frikke, 2008; Nehls & Ketzenberg, 2002). Blue mussels (*Mytilus edulis*) can affect feeding condition of eiders by increasing flesh content and by increasing the size of mussel stocks (Laursen, Møller, Haugaard, Öst, & Vainio, 2019.

The objectives of this study were to test (a) whether intraspecific variation in the size of webbed feet is related to the size of the locomotor apparatus as reflected by wings and its underlying muscular basis in heart and breast muscle; (b) whether the diet is related to the area of the webbed feet, and hence whether the size of the liver as a storage organ increases with the area of the webbed feet. Vitamin E is the main fat‐soluble antioxidant stored in the liver (Møller, Laursen, & Karadas, 2019). Larger webbed feet would imply greater diving ability and hence a reduction in the amount of stored vitamin E (Møller et al., 2019). Finally, (c) if birds with larger webbed feet acquire more food per unit time, this should result in a reduction in the duration of the molting period which again would result in a reduction in the duration of exposure to predators such as white‐tailed sea eagle *Haliaeetus albicilla* that is the main cause of mortality. Viain, Guillemette, and Savard (2015) showed that eiders had lower flight muscle reduction than foot‐propelled diving ducks during molt, apparently as a means of maintenance of the ability to escape from predators.

We tested whether the size of the uropygial gland that is involved in feather maintenance and anti‐microbial activity increases with the area of webbed feet. Because effects of larger webbed feet may be confounded by effects of body size, we included the first principal component as a measure of skeletal body size in the statistical analyses. We used a sample of 233 eiders (*Somateria mollissima*) shot during 2015–2018 in Danish waters during winter (January‐February; *N* = 182) and at prebreeding grounds at Åland, Finland in the central part of the Baltic Sea (April–May; *N* = 51) to test these predictions.

## 2 MATERIALS AND METHODS

### 2.1 Study sites

We obtained a sample of 233 shot eiders specifically collected under license in Denmark during the winter (January–February) 2015–2018 (permit number SN‐302‐009, SNS‐3446‐00103, NST‐3446‐00018, NST‐3465‐00007) and at the Åland Archipelago, Finland during the start of the breeding season April‐May 2016–2017 (permit from Ålands Landskapsregering).

All eiders were sexed according to plumage color (Cramp, 1977). For aging, 1‐ to 2‐year‐old females and females > 2‐year‐olds were classified as calendar year (1, 2 and 3 year classes), and 1‐ to 4‐year‐old males and >4‐year‐old were classified as calendar year (1, 2, 3, 4, and 5 year classes). All specimens were frozen until measurements were taken. We measured flattened wing length and wingspan to the nearest mm. The wing was stretched out so its front leading edge was perpendicular to the edge of a table. The outline of the wing was drawn on a piece of paper that was cut outweighed and compared to a standard piece of 100 cm^2^ to allow for estimation of the area of the wing. The square area between the wings (the so‐called “box area” (Pennycuick, 2008) was calculated as the product of the width of the wing at the base measured with a ruler to the nearest mm and wingspan minus the length of twice the stretched‐out wing to the nearest mm. Body mass was recorded with a balance to the nearest 10 g. Beak length, beak depth, and beak height were measured to the nearest 0.01 mm with a digital calliper (Mitutoyo). From these measurements, we estimated beak volume as 4/3 *πa* ^2^x by where *a* and *b* are the shortest and the longest radii. We measured tarsus length and femur length with a digital calliper to the nearest 0.01 mm (Mitutoyo).

We measured daily growth increments of the second primary by placing it on a piece of white paper under dim light (Grubb, 2006). We measured the distance between five dark and light bands with a digital caliper to the nearest 0.01 mm and then divided this measure by five to obtain an estimate of the average width of a single daily growth increment. We subsequently made a second series of measurements of growth bar width to estimate repeatability of daily growth bar measurements (Falconer & Mackay, 1996). Growth increment measurements were repeatable as shown by measurement of the same feathers twice (*R* = 0.68 [*SE* = 0.05], *F* = 66, 379.57, *df* = 197, 200, *p* < 0.0001). We obtained an index of duration of the molting period by dividing the length of the second primary by the mean value of the two measurements of daily growth bar width.

We removed the right foot for later determination of the area of the stretched out webbed foot. We drew the outline of the foot on a piece of paper that was later weighed on a precision spring balance to the nearest 0.001 g (Gosseron, Cueron, France). This mass was subsequently converted to the nearest cm^2^ by the use of a standard piece of paper with an area of 100 cm^2^. All measurements were made by the same person, and measurements were highly repeatable according to repeated measurements of a subsample.

We extracted the following parts of the body and weighed them to the nearest 0.001 g on a Sartorius precision balance: Breast muscle (*Musculus pectoralis* including *M. p. minor* and *M. p. major*), heart, liver, and gizzard.

The uropygial gland was dissected and weighed on a precision spring balance to the nearest 0.001 g.

Gizzard mass and contents of the gizzard were weighed to the nearest 0.001 g. Gizzard content was separated into nine categories of food items ([*Mytilus edulis*, *Cerastoderma edule*, *Ensis directus*], bivalve spp. [*Littorina littorea*, *Hinia reticulate*, *Buccinum undatum*, *Carcinus maenas*], and other species) under a microscope at a magnification of 10×. A reference collection of benthos species was used for identification (Laursen & Møller, 2016).

Vitamin E was extracted from liver samples as described for fat‐soluble antioxidants (Karadas, Pappas, Surai, & Speake, 2005; Karadas, Surai, & Sparks & N.H.C., 2011; Surai, Speake, Decrock, & Groscolas, 2001). Liver (200–500 mg) was mixed with a 5% solution (w/v in H_2_O) of NaCl (0.7 ml) and ethanol (1 ml) and homogenized for 2 min. Hexane (2 ml) was added, and the mixture was further homogenized for 2 min. The hexane phase containing the carotenoids, Vitamins E and A, and Coenzyme Q_10_ were separated by centrifugation and collected. Extraction with 4 ml hexane was repeated twice, and the extracts were combined, evaporated, and redissolved in a mixture of methanol/dichloromethane (1:1 v/v). Vitamin E (α‐ and γ‐tocopherol) was identified using HPLC (Karadas et al., 2005, 2011; Surai et al., 2001). Samples were injected into a Prominence LC‐20A Series‐HPLC System). Vitamin E was identified using the same HPLC system with a Fluorescence Spectrofluorometer fitted with a Hypersil Gold 5μ C18 reverse‐phase column (10 cm × 4.6 mm; Thermo, US) and a mobile phase of methanol/water (97:3 v/v) at a flow rate of 1.05 ml/min and excitation and emission wavelengths of 295 and 330 nm. A standard solution of α‐tocopherol in methanol was used for calibration.

### 2.2 Statistical analyses

We analyzed variables using generalized linear models (GLM) assuming that variables were normally distributed with an identity link function. We tested for normality using QQ plots and residual plots, but did not find any deviations.

We included the random effects of age, year, country, and age by country as random effects in a model with foot area as a response variable (SAS Institute Inc., 2012).

We used principal component analysis (PCA) on the correlation matrix and the varimax rotation to extract two principal components (SAS Institute Inc., 2012). Five out of eight variables loaded strongly positively on PC1.

We reported mean (*SE*) for summary statistics and estimate (*SE*) in the GLMs. We reported likelihood ratio *χ* ^2^ for testing the significance of the parameter estimates. All analyses were made with JMP (SAS Institute Inc., 2012).

## 3 RESULTS

**Figure 1:**
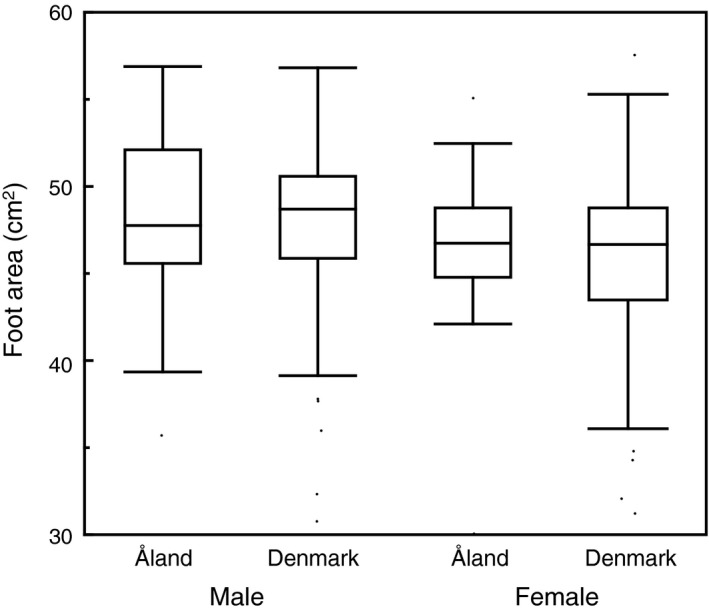
Box plot showing foot area (cm^2^) in relation to country and sex of eiders. Box plots show medians, quartiles, 5‐ and 95‐percentiles and extreme values. Sample size was 233 individuals

The area of webbed feet in 233 adult eiders varied almost twofold between 30 cm^2^ and 58 cm^2^. A random effects model with foot area as the response variable and age, year, country (Figure 1), sex (Figure 1), and country by year interaction only showed a significant difference deviating from zero for year and country hardly accounted for any of the variance (Table 1).

**Table 1:** Random effects model of the relationship between foot area and age, year, country, and country by year interaction

Random effect	Variance ratio	Variance component	*SE*	95% Lower CI	95% Upper CI	Pct. of total variance
Age	0.012	0.242	0.400	−0.542	1.025	0.84
Year	0.000	0.000	0.000	0.000	0.000	0.00
Country	−0.016	−0.319	0.086	−0.487	−0.150	0.00
Country × Year	0.432	8.620	9.684	−10.360	27.599	29.93
Residual		19.939	1.859	16.744	24.150	69.23
Total		28.800	9.900	16.199	64.867	100.00

Note

Sample size was 233 eiders.

**Figure 2:**
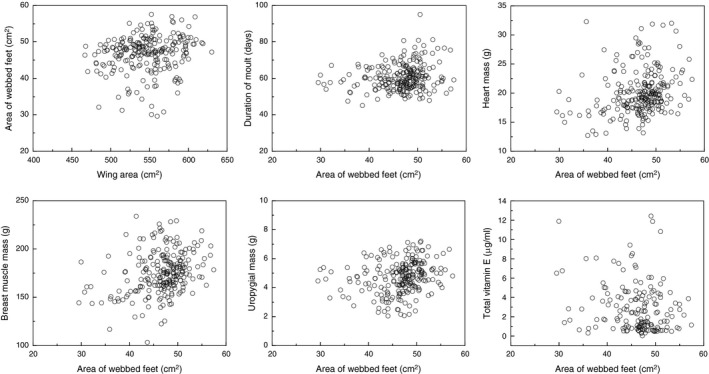
Foot area (cm^2^) in relation to wing area (cm^2^) in adult eiders. Sample size was 233 individuals

A principal component analysis produced two components accounting for 30% and 16% of the variance, respectively (Table 2). Foot area was only weakly correlated with PC1 (*F* = 4.60, *df* = 1, 215, *p* = 0.03, estimate (*SE*) = 0.411 (0.192)) and PC2 (*F* = 3.36, *df* = 1, 215, *p* = 0.07, estimate (*SE*) = −0.532 (0.290)). Wing area increased with foot area (Figure 2; LR *χ* ^2^ = 6.51, *df* = 1, *p* = 0.011, estimate (*SE*) = 0.025 (0.010)), and duration of moult also increased with foot area (LR *χ* ^2^ = 7.49, *df* = 1, *p* = 0.006, estimate (*SE*) = 0.123 (0.045)).

**Table 2:** Principal component analysis on the correlation matrix with Varimax rotation

Eigenvalue	Percent	Cumulative percent	*χ* ^2^	*df*	*p*
2.410	30.125	30.125	644.45	26.729	<0.0001
1.260	15.752	45.962	270.61	22.867	<0.0001

Variable	PC1	PC2
Liver mass	0.275	0.571
Beak volume	0.267	−0.526
Head size	0.663	0.148
Tarsus length	0.501	0.508
Wing length	0.690	−0.312
Feather length	0.716	−0.388
Femur length	0.495	0.351
Uropygial gland	0.582	−0.086

**Figure 3:**
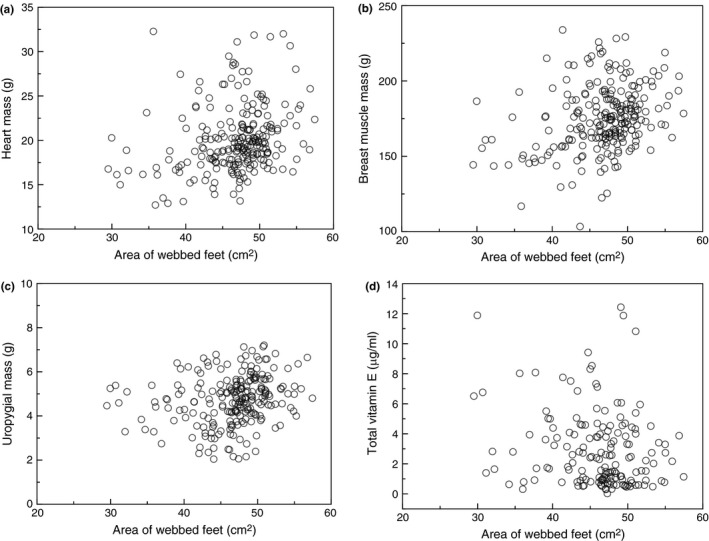
(a) Heart mass (g), (b) breast muscle mass (g), (c) uropygial mass (g), and (d) total vitamin E (μg/ml) in relation to foot area (cm^2^) in adult eiders. Sample size was 233 individuals

Foot area increased with heart mass (Figure 3a; LR *χ* ^2^ = 14.89, *df* = 1, *p* = 0.0001, estimate (*SE*) = 0.331 (0.085)) and with the mass of breast muscles (Figure 3b; LR *χ* ^2^ = 21.80, *df* = 1, *p* < 0.0001, estimate (*SE*) = 0.069 (0.015)).

Foot area increased with beak volume (LR *χ* ^2^ = 4.76, *df* = 1, *p* = 0.029, estimate (*SE*) = 6.192 × 10^5^ (2.082 × 10^5^)). Foot area was significantly correlated with gizzard mass when including all birds from the two sites in the analysis (Figure 3c; LR *χ* ^2^ = 11.13, *df* = 1, *p* = 0.0008, estimate (*SE*) = 0.042 (0.013)). The amount of gizzard content decreased with foot area (LR *χ* ^2^ = 9.66, *df* = 1, *p* = 0.0019, estimate (*SE*) = −0.617 (0.196)). Therefore, eiders with larger webbed feet consumed more cockles and fewer mussels and crabs (Table 3).

**Table 3:** Generalized linear model (GLM) of the relationship between foot area and the abundance of different food items in the gizzard of eiders

Term	Estimate	*SE*	LR *χ* ^2^	*p*
Intercept	47.576	0.347	1,025.95	<0.0001
Cockles	1.083	0.493	4.781	0.029
Mussels	−1.695	0.382	18.884	<0.0001
Beach crabs	−1.362	0.311	18.347	<0.0001

Note

The model had the statistics LR *χ* ^2^ = 40.467, *df* = 3, *p* < 0.0001. Sample size was 233 eiders.

Body condition as reflected by body mass adjusted for femur length increased with foot area (LR *χ* ^2^ = 3.96, *df* = 1, *p* = 0.047, estimate (*SE*) = 0.0050 (0.0025)). The mass of the uropygial gland increased with foot area (LR *χ* ^2^ = 10.65, *df* = 1, *p* = 0.001, estimate (*SE*) = 0.964 (0.292)).

The total amount of vitamin E in liver decreased with foot area of eiders (Figure 3d; LR *χ* ^2^ = 8.03, *df* = 1, *p* = 0.005, estimate (*SE*) = −0.109 (0.038)).

## 4 DISCUSSION

### 4.1 Locomotion and webbed feet

Large webbed feet are likely to facilitate efficient locomotion. If webbed feet contribute to efficient diving, we predicted a positive association between the area of webbed feet and the size of the frontal locomotor apparatus (wing area, heart mass, and breast muscle [e.g., Vágási et al., 2016]). Foot area was positively associated with duration of molt, implying that individuals with larger feet took longer time to finish their molt. Finally, eiders with more efficient locomotion should have elevated body condition as reflected by body mass adjusted for skeletal body size. This was indeed the pattern that we found.

### 4.2 Feeding and webbed feet

Although eiders mainly feed on blue mussels and other bivalves that are easy to catch, individuals with larger webbed feet may be better able to dive deeper and capture mobile prey such as beach crabs, but also stay longer time at the bottom for ingestion of more mussels (Richman & Lovvorn, 2008). For specific food items, we only found a positive relationship between foot area and amount of cockles in the gizzard. Since cockles are the only food items that are buried in the sea bed, we hypothesize that eiders may use the feet to expose them.

Gizzard mass was reduced from the winter grounds in Denmark to the prebreeding area at Åland, due to preparation for breeding (Laursen & Møller, 2016). We found a positive relationship between foot area and gizzard mass both at the wintering and the prebreeding grounds. Individual eiders with large gizzards are able to digest more and larger mussels than individuals with smaller gizzards (Laursen & Møller, 2016), and individual eiders with large gizzards had better body condition at the breeding sites, which increases reproductive potential (Laursen et al., 2019). We expected a positive relationship between foot area and the mass of gizzard content, but surprisingly we found a negative relationship. Gizzard content represents a snapshot and may be influenced by circumstances during the collection of eiders. Most eiders were collected in the morning before they had started feeding. Eiders with large webbed feet had a better body condition, and, therefore, they could have stayed longer at the night roost before flying to the feeding areas in the morning. Thus, eiders may have smaller amounts of food in the gizzard due to a longer rest. However, gizzard mass increased with body mass (Laursen & Møller, 2016), suggesting that a larger gizzard implies a longer period of feeding.

Beak size reflects prey size (Herrel, Podos, Huber, & Hendry, 2005; Hespenheide, 1973; Lederer, 1973; Wilson, 1975). Here, we found that eiders with large beaks also had large webbed feet. This is consistent with the suggestion that large prey are captured by individuals with large beaks and large feet. There was a link between beak morphology used for capture and handling of food and gizzard mass for processing food, eventually giving rise to higher body condition.

Levels of antioxidants in the liver are particularly high in capital breeders that rely on storage for successful incubation and transfer to offspring (Halliwell & Gutteridge, 2007; Surai, 2002). Fat‐soluble antioxidants in the liver of eiders peak at the start of reproduction (Møller et al., 2019). Surprisingly, the total amount of vitamin E in the liver decreased with foot area of eiders. This finding suggests that eiders with large webbed feet have less vitamin E, implying that eiders with large webbed feet worked harder to acquire sufficient amounts of fat‐soluble antioxidants for storage in the liver.

In addition, it is possible that the area of webbed feet will play a role in predator evasion because movement of the feet and the wings combined may push eiders out of reach from a predator such as the white‐tailed sea eagle (*Haliaeetus albicilla*). Finally, we hypothesize that eiders with large feet may reach food items that cannot be reached by eiders with smaller foot area, implying that foot area may play a role in intraspecific competition.

### 4.3 Webbed feet and molt

The duration of molt increased with foot area. This implies that eiders that molted slowly had large feet. Rapid molt could reduce exposure to predators and hence contribute to survival. The use of both feet and wings increased bottom duration considerably compared to dives with feet only (Richman & Lovvorn, 2008). Therefore, it is likely that rapid molt will increase the amount of time at the bottom.

### 4.4 Uropygial glands and webbed feet

The uropygial gland produces fatty secretions that are smeared on the plumage and other surface areas (Moreno‐Rueda, 2017). The function of this gland is disputed although current consensus suggests that it is responsible for elimination of microorganisms from the surface, and/or it is responsible for waterproofing of the plumage (review in Elder, 1954; Jacob & Ziswiler, 1982; Salibián & Montalti, 2009; Moreno‐Rueda, 2017). These are not mutually exclusive hypotheses. Because eiders spend so much time in seawater, either function is possible. Here, we have shown that eiders with larger foot area also have larger relative size of the uropygial gland for their body size. This is consistent with the waterproofing hypothesis since eiders with large foot areas also have a larger anterior locomotor apparatus than eiders with small foot area.

In conclusion, we have shown that the size of webbed feet in a specialist diving duck is functionally linked to locomotion, feeding, and body condition. We hypothesize that the area of webbed feet in other ducks, geese, and swans will prove to have similar function as in eiders, particularly in species that use diving for obtaining food.

## CONFLICT OF INTEREST

None declared.

## AUTHORS CONTRIBUTION

APM developed the study and made the analyses. KL measured the webbed feet. Both authors wrote the paper.

## Data Availability

Data are available at https://doi.org/10.5061/dryad.v34fd09.
